# Copy Number Variation Analysis of 5p Deletion Provides Accurate Prenatal Diagnosis and Reveals Candidate Pathogenic Genes

**DOI:** 10.3389/fmed.2022.883565

**Published:** 2022-07-14

**Authors:** Guoming Chu, Pingping Li, Juan Wen, Gaoyan Zheng, Yanyan Zhao, Rong He

**Affiliations:** ^1^Department of Clinical Genetics, Shengjing Hospital of China Medical University, Shenyang, China; ^2^Department of Obstetrics and Gynecology, Center of Reproductive Medicine, Shengjing Hospital of China Medical University, Shenyang, China; ^3^Center for Medical Genetics and Hunan Key Laboratory of Medical Genetics, School of Life Sciences, Central South University, Changsha, China

**Keywords:** chromosomal translocation, copy number variation, 5p deletion syndrome, 6p duplication, WGS

## Abstract

**Objective:**

5p deletion syndrome, that characterized by cat-like cry and peculiar timbre of voice, is believed to be one of the most common pathogenic copy number variations (CNVs). Variable critical regions on 5p involving a variety of genes contribute to the phenotypic heterogeneity without specific correlation. The objective of this study was to examine the genotype–phenotype correlation of 5p deletion syndrome, and to redefine 5p deletion syndrome relevant regions. In addition, we demonstrate the potential use of whole genome sequencing (WGS) to identify chromosomal breakpoints in prenatal diagnosis.

**Methods:**

Three families with women undergoing prenatal diagnosis and two children were recruited. Karyotyping, CNV-seq, fluorescence *in situ* hybridization, WGS, and Sanger sequencing were performed to identify the chromosomal disorder.

**Results:**

We reported three families and two children with CNVs of 5p deletion or combined 6p duplication. Five different sizes of 5p deletion were detected and their pathogenicity was determined, including 5p15.33-p15.31 [1–7,700,000, family1-variant of uncertain significance (VUS)], 5p15.33 (1–3,220,000, family 2-VUS), 5p15.33-p15.31 (1–7,040,000, family 3-VUS), 5p15.33-p15.31 (1–8,740,000, child 1-pathogenic) and 5p15.31-p15.1 (8,520,001–18,080,000, child 2-pathogenic). One duplication at 6p25.3-p24.3 (1–10,420,000) was detected and determined as likely pathogenic. The chromosomal breakpoints in family 3 were successfully identified by WGS.

**Conclusion:**

Some critical genes that were supposed to be causative of the symptoms were identified. Relevant region in 5p deletion syndrome was redefined, and the chr5:7,700,000–8,740,000 region was supposed to be responsible for the cat-like cry. The great potential of WGS in detecting chromosomal translocations was demonstrated. Our findings may pave the way for further research on the prevention, diagnosis, and treatment of related diseases.

## Introduction

5p deletion syndrome, first described by Lejeune et al. in 1963 (1), is a rare genetic disease that defined as variable-sized deletions of the short arm of chromosome 5. 5p deletion syndrome is one of the most common pathogenic copy number variations (CNVs), with a reported incidence of 1 per 15,000–50,000 live births (2). Typical features of this disorder are post-natal cat-like cry and a peculiar timbre of voice. Other notable features include developmental delay, severe psychomotor and intellectual disability, rounded face, hypertelorism, epicanthic folds, and cardiac, cerebral, renal and gastrointestinal malformations (3). Ultrasound is not recommended for *in utero* diagnosis of 5p deletion syndrome owing to the non-specific ultrasonographic features (4), which underscores the importance of genetic prenatal diagnosis. Southern Blot was first used for the diagnosis of 5p deletion syndrome in 1989 (5). However, due to the limited coverage of probes, Southern Blot is unable to detect all 5p regions and cannot identify the breakpoint. Karyotyping has been the gold-standard in prenatal diagnosis for decades (6). However, detection of 5p microdeletion is beyond the capability of this technique (7). Fluorescence *in situ* hybridization (FISH) requires *a priori* knowledge and it is difficult to detect all microdeletions and microduplications due to the limitations of designed probes (8). Chromosomal microarray analysis (CMA) and copy number variation sequencing (CNV-seq) are reliable and accurate prenatal techniques for identifying CNVs. The latter technique has the ability to identify cryptic CNVs located in regions with insufficient probe coverage on CMA platforms, and it also has a higher sensitivity for detecting low-level mosaicism (9). Phenotypic heterogeneity in different cases of 5p deletion syndrome depends on the specific breakpoints on 5p that involve a variety of genes. All the above-mentioned techniques have shortcomings in detecting the specific breakpoints. Whole genome sequencing (WGS), on the contrary, has the potential to define chromosomal breakpoints at nucleotide-pair resolution (10). WGS combined with Sanger sequencing perform better in detecting chromosomal translocations compared with FISH and karyotyping, show higher resolution and the ability to identify the breakpoint, and may replace traditional cytogenetic methods in the diagnosis of balanced translocation (11, 12).

Herein, we reported three families and two children with CNVs of 5p deletion. The objective of the present study was to assess the genotype-phenotype correlation of 5p deletion syndrome, and redefine the relevant region of 5p deletion syndrome. In addition, we underscored the potential use of WGS for identifying chromosomal breakpoints in prenatal diagnosis.

## Case Presentation

**FIGURE 1:**
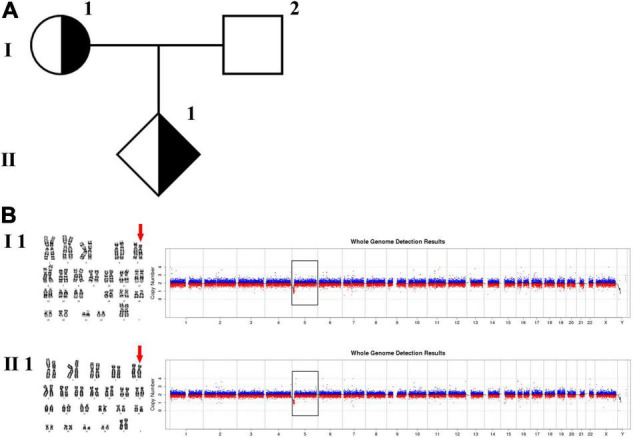
Karyotyping and CNV-seq results of family 1. **(A)** The pedigree of the family 1. **(B)** Karyotyping and CNV-seq results of the pregnant woman (I-1) and amniotic fluid (II-1). The red arrow indicates the deletion of 5p in karyotyping results. The box indicates the deletion of 5p in CNV-seq results.

In family 1 (Figure 1 and Table 1), the pregnant woman (I-1) was 19^+5^ weeks of gestation and accepted getting genetic counseling for a history of spontaneous miscarriage. Both the woman (I-1) and her husband (I-2) were phenotypically normal. Amniocentesis was performed due to advanced maternal age, and karyotyping and CNV-seq of the fetus and couples were conducted.

**TABLE 1:** Summary of samples included in our study.

Sample no.	Age	Karyotype (Chromosome G-banding)	CNV-seq result	Clinical significance and evidence of CNV (ACMG and ClinGen)	Clinical features
Family 1(I-1)	41 years	46,XX,del(5) (p15.3)	seq[GRCh37] 5p15.33p15.31(1_7700000) × 1	VUS; 0.45 points (3B: number of protein-coding RefSeq genes included in the CNV is between 25 and 34, 0.45 points)	Normal
Family 1(I-2)	40 years	46,XY	Normal	–	Normal
Family 1(II-1)	fetus 19^+5^ weeks	46,XX,del(5) (p15.3)	seq[GRCh37] 5p15.33p15.31(1_7700000) × 1	VUS; 0.45 points (3B: number of protein-coding RefSeq genes included in the CNV is between 25 and 34, 0.45 points)	Normal
Family 2(II-2)	60 years	46,XX,t(Y;5) (q11.23;p15.3)	Normal	–	Normal
Family 2(II-1)	35 years	46,X,der(Y) (Ypter→q11.23:5p15.3→pter)	seq[GRCh37] 5p15.33(1_3220000) × 3	VUS; 0 point (3A: number of protein-coding RefSeq genes included in the CNV is less than 24, 0 point)	Normal
Family 2(II-2)	30 years	46,XX,der(5) (Yqter→q11.23:5p15.3→qter)	seq[GRCh37] 5p15.33(1_3220000) × 1	VUS; 0 point (3A: number of protein-coding RefSeq genes included in the CNV is less than 24, 0 point)	Normal
Family 2(III-1)	7 years	46,XX,der(5) (Yqter→q11.23:5p15.3→qter)	seq[GRCh37] 5p15.33(1_3220000) × 1	VUS; 0 point (3A: number of protein-coding RefSeq genes included in the CNV is less than 24, 0 point)	Normal
Family 2(III-2)	Fetus 19^+2^ weeks	46,XX,der(5) (Yqter→q11.23:5p15.3→qter)	seq[GRCh37] 5p15.33(1_3220000) × 1	VUS; 0 point (3A: number of protein-coding RefSeq genes included in the CNV is less than 24, 0 point)	Normal
Family 3 (III-2)	48 years	46,XY	Normal	–	Normal
Family 3(IV-2)	22 years	46,XX	seq[GRCh37] 5p15.33p15.31(1_7040000) × 1	VUS; 0.45 points (3B: number of protein-coding RefSeq genes included in the CNV is between 25 and 34, 0.45 points)	hearing loss; mild mental retardation
			seq[GRCh37] 6p25.3p24.3(1_10420000) × 3	likely pathogenic; 0.90 points (3B: number of protein-coding RefSeq genes included in the CNV is between 25 and 34, 0.45 points; 4C: the reported phenotype is consistent with the gene/genomic region, but not highly specific and/or with high genetic heterogeneity, 0.15 points; 5H: the patient phenotype is highly specific and consistent with what has been described in similar cases, 0.30 points)	
Family 3(IV-3)	13 years	46,XX	seq[GRCh37] 5p15.33p15.31(1_7040000) × 1	VUS; 0.45 points (3B: number of protein-coding RefSeq genes included in the CNV is between 25 and 34, 0.45 points)	deafness; moderate mental retardation; walking disorder
			seq[GRCh37] 6p25.3p24.3(1_10420000) × 3	likely pathogenic; 0.90 points (3B: number of protein-coding RefSeq	
				genes included in the CNV is between 25 and 34, 0.45 points; 4C: the reported phenotype is consistent with the gene/genomic region, but not highly specific and/or with high genetic heterogeneity, 0.15 points; 5H: the patient phenotype is highly specific and consistent with what has been described in similar cases, 0.30 points)	
Family 3(V-1)	fetus 22 weeks	46,XX	seq[GRCh37] 5p15.33p15.31(1_7040000) × 1	VUS; 0.45 points (3B: number of protein-coding RefSeq genes included in the CNV is between 25 and 34, 0.45 points)	–
			seq[GRCh37] 6p25.3p24.3(1_10420000) × 3	likely pathogenic; 0.90 points (3B: number of protein-coding RefSeq genes included in the CNV is between 25 and 34, 0.45 points; 4C: the reported phenotype is consistent with the gene/genomic region, but not highly specific and/or with high genetic heterogeneity, 0.15 points; 5H: the patient phenotype is highly specific and consistent with what has been described in similar cases, 0.30 points)	
Child 1	3 months	–	seq[GRCh37] 5p15.33p15.31(1_8740000) × 1	pathogenic; 1.05 points (3C: number of protein-coding RefSeq genes included in the CNV is more than 35, 0.90 points; 5A: Observed copy number loss is *de novo*, 0.15 points)	cat-like cry; developmental retardation
Child 2	2 years	–	seq[GRCh37] 5p15.31p15.1 (8520001_18080000) × 1	pathogenic; 1.30 points (2A: complete overlap of an established HI gene *TRIO* [ID: ISCA-25787], 1.00 point; 5A: Observed copy number loss is *de novo*, 0.30 points)	hypotonia; dysplasia of corpus callosum; developmental retardation

**FIGURE 2:**
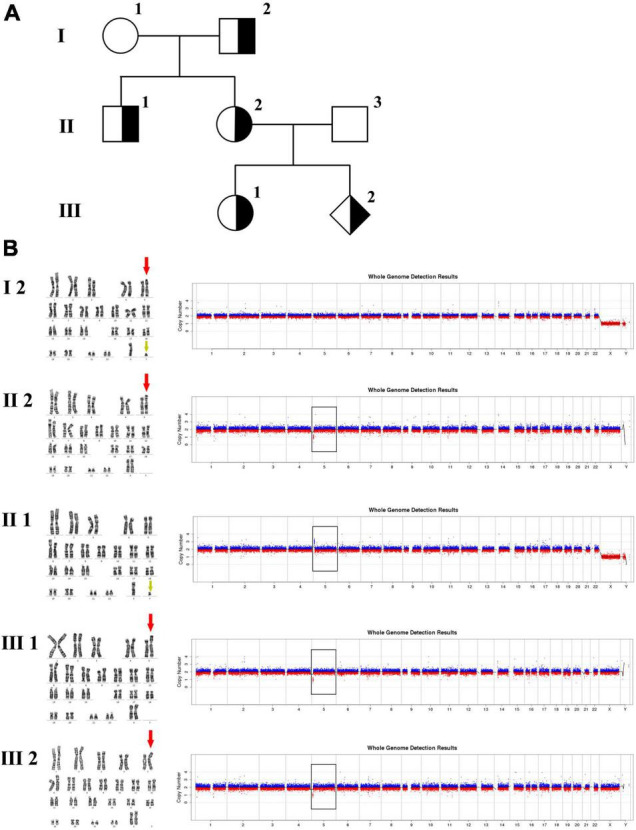
Karyotyping and CNV-seq results of family 2. **(A)** The pedigree of the family 2. **(B)** Karyotyping and CNV-seq results. The red arrow indicates the derived chromosome 5 in karyotyping results, and the yellow arrow indicates the derived chromosome Y. The box indicates the deletion or duplication of 5p in CNV-seq results.

In family 2 (Figure 2 and Table 1), the pregnant woman (II-2) was 19^+2^ weeks of gestation, obtaining getting genetic counseling for positive results by non-invasive prenatal testing (NIPT). NIPT was performed on Illumina Nextseq CN500 platform, the quantitated DNA library was sequenced using the single–ended 36 bp sequencing protocol. NIPT result presented a fetal fraction of 12.26%, indicated a 3.20 Mb deletion at 5p15.33:100,000–3,299,999 (z score: –10.212), and showed two chromosome X plus a abnormal chromosome Y which only retained a 0.32 Mb fragment at Yq11.23:28,460,000-28,780,000 (Z score: 6.911). All the other individuals were phenotypically normal. Amniocentesis was performed followed by karyotyping and CNV-seq of the fetus and selected family members (I-2, II-1, II-2, III-1, and III-2). In addition, FISH was conducted on the fetus and selected family members (I-2, II-1, II-2, and III-2).

In family 3 (Figure 4 and Table 1), the pregnant woman (IV-2) was 22 weeks of gestation, receiving genetic counseling for hearing loss and mild intellectual disability. IV-3 was a girl with the phenotype of congenital deafness, moderate intellectual disability, and walking disorder. All the other individuals were phenotypically normal. Amniocentesis was performed followed by karyotyping and CNV-seq of the fetus and family members (III-2, IV-2, IV-3, and V-1). In addition, WGS was carried out on III-2.

Child 1 was a boy with the phenotype of cat-like cry and developmental delay. Both his parents were phenotypically normal. CNV-seq of the family members was conducted.

Child 2 was a girl with the phenotype of hypotonia, dysplasia of corpus callosum, and developmental delay. Both her parents were phenotypically normal. CNV-seq of the family members was conducted.

This study was approved by the Medical Ethics Committee of the Chinese PLA General Hospital (approval number S2016-120-02) and written informed consent was obtained from the family members.

## Materials and Methods

The protocol for diagnostic work up for 5p deletion syndrome was shown in Supplementary Figure 1.

### Chromosome G-Banding

Amniotic fluid cells were cultured in medium (#99473, Irvine Scientific, United States) for one week. Lymphocytes isolated from peripheral blood were cultured in medium for three days. G-banding karyotyping was carried out on cultured amniotic fluid cells and lymphocytes using standard techniques. Six metaphase cells were analyzed, and 20 metaphase cells were counted by two examiners based on the principle of double-blinding. The karyotyping results were determined according to the International System for Human Cytogenetic Nomenclature 2016.

### CNV-Seq

Genomic DNA (gDNA) was extracted from amniotic fluid and peripheral blood samples using the Genomic DNA extraction kit (QIAGEN, Germany) according to the manufacturer’s instructions. The concentration of gDNA was measured using the Invitrogen Qubit 2.0 (ThermoFisher Scientific). The library was constructed using Library construction kit (#KR2000, Berry Genomics, Beijing, China). DNA library was quantitated using the KAPA library quantification kits (#KK4824, Roche). The quantitated DNA library was sequenced on Illumina Nextseq CN500 platform, and the sequencing data was were analyzed by data analysis system (Berry Genomics, Beijing, China).

### Fluorescence *in situ* Hybridization

Fluorescence *in situ* hybridization was performed to confirm the chromosomal translocations. Bacteria artificial chromosome (BAC) clones (Illumina) including RP11-263C17 (mapped to Yq11.23), RP11-1011C7 (mapped to 5q22.3), RP11-91E18 (mapped to 5q13.2-q13.3), and RP11-846K3 (mapped to 5p15.33) were used as FISH probes on metaphase chromosomes. All FISH procedures were performed according to the manufacturer’s instructions. The results were analyzed and documented using CytoVision system (Leica Microsystems).

### Whole Genome Sequencing and Sanger Sequencing

Genomic DNA was measured using agarose gel electrophoresis for analyzing degradation and excluding RNA contamination. The purity and concentration of gDNA were measured using Qubit 2.0. Qualified DNA was fragmented to 350 bp by sonication (Covaris Inc., Woburn, MA, United States). Library was constructed using NEBNext Ultra DNA Library Prep Kit for Illumina (#E7370, New England Biolabs, Ipswich, MA, United States) according to the manufacturer’s instructions, including end repair, A-tailing, adaptor ligation, cleanup of adaptor-ligated DNA, PCR enrichment of adaptor-ligated DNA, and cleanup of PCR reaction. Concentration of the library was measured by quantitative PCR. Paired-end sequencing with 150 bp was carried out on the Illumina platform. The data were mapped to the reference genome (hg19, GRCh37) using the software BWA.^1^ Reads were sorted with SAMTools. SNVs and InDels were defined using the Genome Analysis Toolkit.^2^ SVs were defined using BreakDancer. Chromosomal breakpoints were identified based on the WGS results and verified by Sanger sequencing. Sanger sequencing was performed on 3730 DNA Analyzer (Applied Biosystems, United States) using BigDye™ Terminator v3.1 Cycle Sequencing Kit (Applied Biosystems, United States).

### Quantitative Fluorescent PCR for Short Tandem Repeat Detection

Quantitative fluorescent PCR (QF-PCR) was performed to determine the copy number of chromosomes X and/or Y. The selected short tandem repeat (STR) polymorphisms were located on chromosomes X and/or Y (AMEL, XHPRT, X22, DXS6803, DYS448, TAF9L, DXYS267, and SRY). STR amplification was performed using PrimeSTAR^®^ HS DNA polymerase (Takara, Dalian, China). Fragment analysis was performed on the ABI3730 DNA sequencer (Applied Biosystems, Foster City, CA, United States).

## Results

### Family 1

The phenotypically normal couple received genetic counseling due to a history of miscarriage, and amniocentesis was performed for the 41-year-old pregnant woman. The pedigree is shown in Figure 1A. As shown in Figure 1B, the karyotyping results of the amniotic fluid (II-1) and the pregnant woman (I-1) were 46,XX,del(5) (p15.3). CNV-seq results also showed a 7.70 Mb deletion at 5p15.33-p15.31 (1–7,700,000) in both pregnant woman and the fetus. According to technical standards recommended by the American College of Medical Genetics and Genomics (ACMG) and the Clinical Genome Resource (ClinGen) (13), the variation was considered as variant of uncertain significance (VUS) (Table 1). Therefore, the pregnancy was suggested to continue as the fetus carried the same CNV as the mother without aberrant phenotype. Finally, a healthy baby with normal weight and length was delivered at 39 weeks of gestation.

### Family 2

**FIGURE 3:**
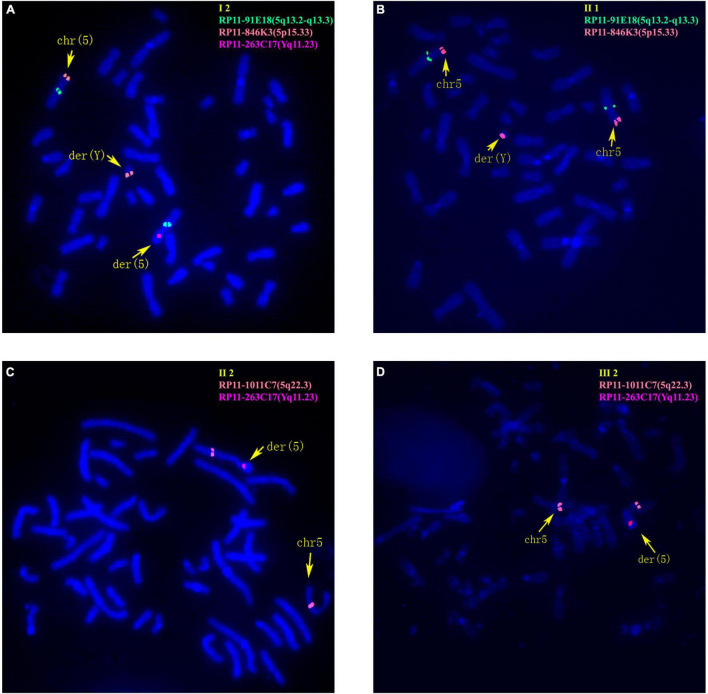
Fluorescence *in situ* hybridization (FISH) results of family 2. **(A)** FISH result of metaphase cells from I-2 with BAC probes of RP11-91E18 showing signals mapped to 5q13.2-q13.3 (green), RP11-846K3 showing signals mapped to 5p15.33 (orange) and RP11-263C17 showing signals mapped to Yq11.23 (red). **(B)** FISH result of metaphase cells from II-1 with RP11-91E18 that are mapped to 5q13.2-q13.3 (green) and RP11-846K3 that are mapped to 5p15.33 (orange). **(C)** FISH result of metaphase cells from II-2 with BAC probes of RP11-1011C7 showing signals mapped to 5q22.3 (orange) and RP11-263C17 mapped to Yq11.23 (red). **(D)** FISH result of metaphase cells from III-2 with RP11-1011C7 showing signals mapped to 5q22.3 (orange) and RP11-263C17 mapped to Yq11.23 (red).

The 30-year-old pregnant woman came for genetic counseling due to positive NIPT results and corresponding genetic tests were conducted for the family members and the fetus. The pedigree is shown in Figure 2A. As shown in Figure 2B, the karyotyping results of the amniotic fluid (III-2), the pregnant woman (II-2), and the first child (III-1) were 46,XX,der(5) (Yqter→q11.23:5p15.3→qter). The CNV-seq results showed a 3.22 Mb deletion at 5p15.33 (1–3,220,000), but no sequence from chromosome Y was detected. The pregnant woman’s father (I-2) showed a karyotype of 46,X,t(Y;5) (q11.23;p15.3), and the CNV-seq result was normal. The pregnant woman’s brother (II-1) carried an abnormal chromosome Y according to the karyotyping result of 46,X,der(Y) (Ypter→q11.23:5p15.3→pter), while the CNV-seq result showed a 3.22 Mb duplication at 5p15.33 (1–3,220,000) with absence of abnormality in chromosome Y. As shown in Figure 3, FISH results showed a balanced translocation between chromosome Yq and 5p in I-2. II-1 and II-2 inherited the derived Y and derived 5 from their father (I-2), respectively. III-2 carried the same derived 5 from II-2. According to the ACMG & ClinGen technical standards, 5p15.33 (1–3,220,000) deletion and duplication were classified as VUS (Table 1). QF-PCR results showed three peaks at the locus X22 (Supplementary Figure 2). Thus, the fetus was judged as normal with the inherited CNV from the phenotypically normal mother. Finally, the baby was delivered at 39^+2^ weeks of gestation with appropriate weight and length.

**FIGURE 4:**
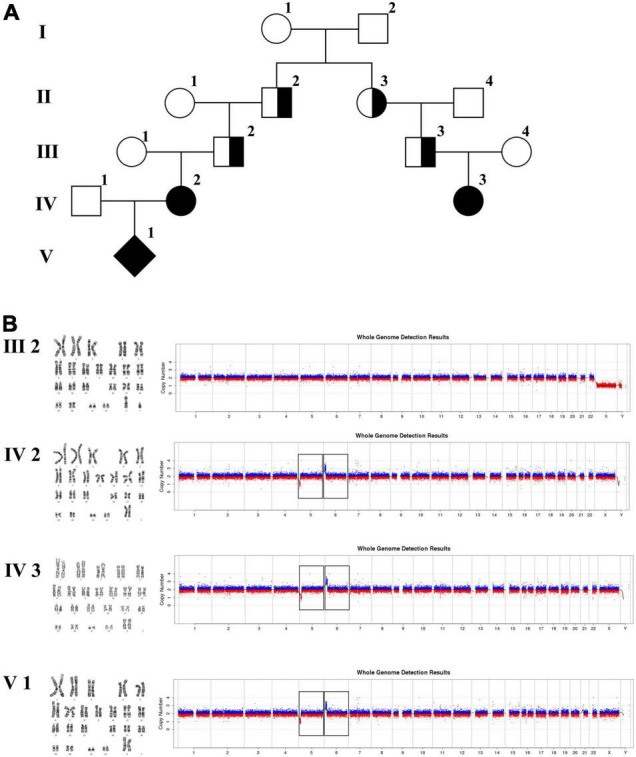
Karyotyping and CNV-seq results of family 3. **(A)** The pedigree of the family 3. **(B)** Karyotyping and CNV-seq results. The box indicates the deletion of 5p and duplication of 6p in CNV-seq results.

### Family 3

**FIGURE 5:**
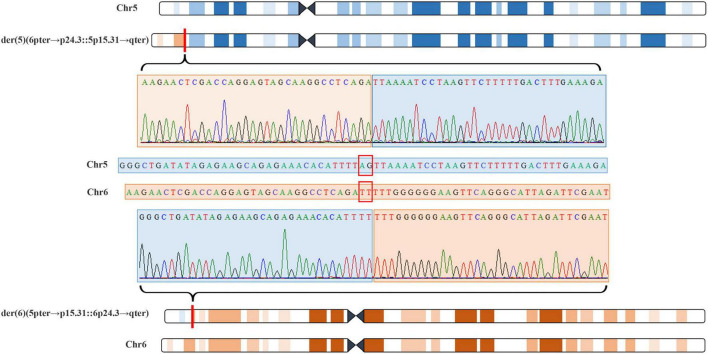
Whole genome sequencing (WGS) and Sanger sequencing identified balanced translocation of pregnant woman’s father (III-2). Structure sketch map of chromosome 5 (blue) and chromosome 6 (yellow) are shown at the top and bottom. The Sanger sequencing results across the breakpoints on chromosome 5 and chromosome 6 are displayed, respectively, in the middle. Red box indicates 2-bp deletion in the breakpoint junction on chromosome 5 and chromosome 6.

The 22-year-old pregnant woman received genetic counseling for hearing loss and mild intellectual disability as well as a disabled cousin. The pedigree is shown in Figure 4A. As shown in Figure 4B, karyotypes of the amniotic fluid (V-1) and other individuals were normal. The CNV-seq results of amniotic fluid (V-1) showed a 7.04 Mb deletion at 5p15.33-p15.31 (1–7,040,000) and a 10.42 Mb duplication at 6p25.3-p24.3 (1–10,420,000) (Figure 4B and Table 1). The CNV-seq results of the pedigree analysis indicated that the pregnant woman (IV-2) and another family member (IV-3) carried the same deletion and duplication, and the other CNV-seq results from the remaining family members were normal. The WGS and Sanger sequencing result of the pregnant woman’s father (III-2) showed a balanced translocation between chromosomes 5p and 6p (Figure 5), and we determined the karyotype of III-2 as 46,XY,t(5;6) (p15.31;p24.3) by WGS and Sanger sequencing. By analyzing WGS and Sanger sequencing data, we found the precise translocation breakpoints located at chr5:7,047,736–7,047,736,739 and chr6:10,418,611–10,418,614, respectively. There were both 2-bp deletion in the breakpoint junction at chr5:7,047,737–7,047,738 (AG) and chr6:10418612-10418613 (TT), respectively. According to the ACMG & ClinGen technical standards, the CNV 5p15.33-p15.31 (1–7,040,000) deletion was classified as VUS; the CNV 6p25.3-p24.3 (1–10,420,000) duplication was considered “likely pathogenic.” Thus, the family was counseled that the fetus may show the same symptoms such as hearing loss and varying degrees of mental retardation as the mother and IV-3. Finally, the family opted for termination of pregnancy.

### Child 1

Child 1 was a 3-month-old boy with phenotypes of cat-like cry and developmental delay. CNV-seq result of Child 1 showed an 8.74 Mb deletion at 5p15.33-p15.31 (1–8,740,000) (Table 1), while the CNV-seq results of his parents were normal. According to ACMG & ClinGen technical standards, the variation was considered “pathogenic”.

### Child 2

Child 2 was a 2-year-old girl with phenotypes of hypotonia, dysplasia of corpus callosum, and developmental delay. CNV-seq result of Child 2 showed a 9.56 Mb deletion at 5p15.33-p15.31 (8,520,001–18,080,000) (Table 1), while the CNV-seq results of her parents were normal. The variation covering dosage sensitivity gene *TRIO* (Haploinsufficiency Score: 3) was considered “pathogenic.”

## Discussion

As common CNVs, 5p partial deletions were reported to be usually *de novo* and caused by paternal translocation in 80–90% of cases, possibly arising from chromosome breakage during gamete formation of males (2). Approximately 10–15% of 5p partial deletions were attributed to balanced parental translocation (14). Here, in family 2, the karyotyping and FISH results demonstrated that the translocation between Yq11.23 and 5p15.33 was the causal lesion. In family 3, both CNV-seq and WGS results showed that the translocation between 5p15.33-p15.31 and 6p25.3-p24.3 was the reason for the CNVs of 5p deletion combined 6p duplication. No translocation was detected in family 1, and the variation was supposed to arise from chromosomal breakage during gamete formation.

The observed variability of phenotypes among individuals with 5p deletion is attributed to the differences of the breakpoints. Studies have demonstrated the correlation between deletion size and clinical features. For example, 5pter-p15.32 is associated with speech delay, the proximal part of 5p15.3 and 5p15.2 may be responsible for the cat-like cry, and deletion of the proximal part of 5p15.2 underlies the typical facial dysmorphism (15, 16). In our study, we report three prenatal diagnosis pedigrees and two children. In these families, 5p deletions with variable sizes were detected in related individuals. However, the pregnant woman in family 1 who carried the 5pter-p15.31 variation was apparently normal, and the fetus (II-1) showed no symptoms after birth. Despite a few reports describing its relation with dyskeratosis congenita, *TERT* gene is currently only confirmed to be associated with shortened telomeres, and cannot explain all characteristics of the 5p deletion syndrome (17, 18). Hence, according to the ClinGen database and our report, we inferred that the 5pter-p15.31 region (1–7,700,000) may not involve any dosage sensitive genes, and the monosomy of inner 5p15.33 (1–3,220,000) in family 2 and 5pter-p15.31 (1–7,080,000) in family 3 were not pathogenic. Similar cases have been reported previously wherein individuals who carried a 5p terminal (1–4,500,000) deletion and an interstitial deletion at 4,200,304–7,081,712 showed no phenotypic effects (16, 19). Zhang et al. reported a 5p (17,630–4,805,799) deletion pedigree in which the proband showed poor feeding, bradycardia, hoarse cry due to transient bilateral vocal cord dysfunction, motor and language retardation, and specific facial features. The father and sister of the proband were carriers of the same 5p deletion and displayed learning difficulties and behavioral issues, but with no dysmorphic features. However, another sister and brother of the proband displayed behavioral disorder with a normal chromosome 5 (20); therefore the pathogenic cause of affected family members should not be the 5p deletion. Hence, we have always stressed the importance of parental origin verification in the clinical interpretation of fetal CNVs. Shi et al. verified the parental origin of 141 fetal CNVs and identified that 102 CNVs (72.3%) were derived from the parents, with 74 CNVs of likely benign clinical significance (21). In contrast to the benign 5p (1–7,700,000) deletion described by us in family 1, the child 1 carries a 1.04 Mb longer deletion of 5p15.33-p15.31 (1–8,740,000), and showed a high pitched, monotonous cry. This indicates that the 1.04 Mb (chr5:7,700,000–8,740,000) region may be responsible for the cat-like cry, which is more precise than the previously reported cat-like cry critical region. By searching the DatabasE of genomiC varIation and Phenotype in Humans using Ensembl Resources (DECIPHER) and ClinGen database, we identified only four protein-coding genes involved in this region with no reported dosage sensitivity. These include *ADCY2*, which partially overlaps the deletion, and the complete sequences of *C5orf49*, *FASTKD3*, and *MTRR*. *ADCY2* gene encodes a member of the family of adenylate cyclases, which are membrane-associated enzymes that catalyze the formation of the secondary messenger cyclic adenosine monophosphate (cAMP) in response to G-protein signaling (22). The *C5orf49* gene function is not well characterized. Monika et al. reported that *C5orf49* gene encodes a protein localized to cilia and has ciliary functions (23). *FASTKD3* gene encodes a member of a small family of Fas-activated serine/threonine kinase domain (FASTKD) containing proteins, which is required for normal mitochondrial respiration by enhancing MT-CO1 mRNA translation and mitochondrial complex IV assembly and activity (24, 25). *MTRR* gene encodes a member of the ferredoxin-NADP (+) reductase (FNR) family of electron transferases, which is responsible for the reactivation of methionine synthase (MTR/MS) activity by catalyzing the reductive methylation of MTR-bound cob (II) alamin (26). *MTRR* gene has been shown to be associated with Homocystinuria-Megaloblastic Anemia, Cble Complementation Type and Neural Tube Defects, Folate-Sensitive. These findings will be helpful for genotype-phenotype correlation analysis of 5p deletion syndrome.

6p duplication in most patients results from the unbalanced inheritance of a parental translocation, along with deletion of another chromosome (27). The clinical symptoms of these patients are caused by 6p duplication combined with the effect of another chromosome abnormality (28). Since the breakpoints of 6p vary from 6p11 to 6p25, 6p duplication also presents variable clinical characteristics of pre- or post-natal growth restriction with a short final stature, microcephaly, distinct facial features, developmental delay, congenital heart defects, sensorineural hearing impairment, renal complications, speech delay, and intellectual disability (27, 29). A search of the DECIPHER database revealed four patients carrying 6p duplications (patient numbers 284585, 393057, 392292, and 392965) with no other chromosome abnormalities, and they displayed variable phenotypic features including global developmental delay, cryptorchidism, unilateral renal agenesis, abnormality of the thyroid gland, short stature, and microcephaly. All patients showed multisystem anomalies. Additionally, 6p25 duplication may be associated with ocular developmental abnormalities and glaucoma (30). There is also phenotypic heterogeneity among family members with the same 6p25 duplication. For instance, Fontana et al. reported a family with four members carrying a 6p25.3-p25.2 duplication, and all four family members displayed brachydactyly with sporadic cardiac abnormality, hypothyroidism, and special facial features (31). Consistently, the two affected individuals of family 3 in this report also displayed different phenotypes. The pregnant woman (IV-2) showed symptoms of hearing loss and mild intellectual disability, while her younger female cousin (IV-3) had congenital deafness, moderate intellectual disability, and walking problems. The 6p25.3-p24.3 duplication region in family 3 includes 44 protein-coding genes. Of these, 13 genes are included in the Online Mendelian Inheritance in Man database as disease-causing genes (shown in Table 2). The pathogenicity of altered gene dosage remains to be determined. Five out of those 13 genes were reported to be associated with hearing loss or intellectual disability, including *forkhead box C1* (*FOXC1*), *serpin family B member 6* (*SERPINB6*), *tubulin beta 2A class IIa* (*TUBB2A*), *tubulin beta 2B class IIb* (*TUBB2B*) and *anscription factor AP-2 alpha* (*TFAP2A*). *FOXC1* was reported as a haploinsufficiency sensitive gene. *FOXC1* mutations can lead to Axenfeld-Rieger syndrome which is characterized by developmental defects of the anterior chamber of the eye, maxillary hypoplasia, hypodontia, microdontia, umbilical abnormalities, and sensorineural deafness (32). *SERPINB6* gene is associated with deafness, featured as progressive, age-related sensorineural hearing loss (33). Defects of *TUBB2A* and *TUBB2B* genes are linked to cortical dysplasia in combination with other brain malformations type 5 and type 7, respectively. The clinical manifestations include cortical dysplasia, hypoplasia of the corpus callosum, global developmental delay, and seizures (34). Davies et al. suggested the potential involvement of *TFAP2A* in the development of the anterior eye chamber (35). Tekin et al. identified a heterozygous deletion/insertion mutation in the *TFAP2A* gene in a 4-year-old Turkish girl with Branchiooculofacial syndrome which is characterized by branchial cleft sinus defects, ocular anomalies such as microphthalmia and lacrimal duct obstruction, and a dysmorphic facial appearance (including cleft or pseudocleft lip/palate). In addition, the girl showed the phenotype of sensorineural hearing loss (36). Here in family 3, we performed WGS and Sanger sequencing assays, and identified a breakpoint of chromosome 6 (chr6:10,418,611-10,418,614), which is located in the intron 1 of *TFAP2A* (NM_001042425). Further study of the involved genes in 6p25.3-p24.3 is essential for elucidating the pathogenesis of 6p25.3-p24.3 duplication.

**TABLE 2:** Summary of OMIM genes in 6p25.3p24.3(1_10420000).

Gene symbol	OMIM ID	Disease (OMIM ID)	Inheritance	Phenotype
*BLOC1S5* biogenesis of lysosomal organelles complex 1 subunit 5	607289	Hermansky-Pudlak syndrome 11 (619172)	AR	Ocular skin albinism
*DSP* desmoplakin	125647	Arrhythmogenic right ventricular dysplasia 8 (607450)	AD	Arrhythmias
		Cardiomyopathy, dilated, with woolly hair and keratoderma (605676)	AR	Generalized palmoplantar epidermal striate keratosis, hair curl and left ventricular dilated cardiomyopathy
		Dilated cardiomyopathy with woolly hair, keratoderma, and tooth agenesis(615821)	AD	Bilateral dilated cardiomyopathy, skin hyperkeratosis, hair curl, palmoplantar skin keratosis and oligodontia
		Epidermolysis bullosa, lethal acantholytic (609638)	AR	Generalized oozing erosion of the entire skin
		Keratosis palmoplantaris striata II (612908)	AD	Thickening of palms and soles of feet, flexion of fingers
		Skin fragility-woolly hair syndrome (607655)	AR	fragile skin with blistering, focal and diffuse palmoplantar keratosis, keratotic plaques on the trunk and limbs, woolly hair and varying degrees of alopecia
*F13A1* coagulation factor XIII A chain	134570	Factor XIIIA deficiency (613225)	AR	Hemorrhage
*FARS2* phenylalanyl-tRNA synthetase 2, mitochondrial	611592	Combined oxidative phosphorylation deficiency 14 (614946)	AR	Growth retardation, intractable epilepsy, lactic acidosis
		Spastic paraplegia 77, autosomal recessive (617046)	AR	Lower limb spasm in early childhood, gait difficulty
*FOXC1* forkhead box C1	601090	Anterior segment dysgenesis 3, multiple subtypes (601631)	AD	Iris dysplasia, anterior chamber angle dysplasia, juvenile glaucoma
		Axenfeld-Rieger syndrome, type 3 (602482)	AD	Developmental defects of the anterior chamber of the eye, maxillary hypoplasia, hypodontia, microdontia, umbilical abnormalities and sensorineural deafness
*IRF4* interferon regulatory factor 4	601900	[Skin/hair/eye pigmentation, variation in, 8] (611724)	–	Brown hair, freckles, fair skin, blue or light eyes, sensitive to ultraviolet rays
*LYRM4* LYR motif containing 4	613311	Combined oxidative phosphorylation deficiency 19 (615595)	AR	Dyspnea, hypotonia, lactic acidosis, gastroesophageal reflux
*NQO2* N-ribosyldihydronicotinamide: quinone reductase 2	160998	{?Breast cancer susceptibility} (114480)	AD, SMu	Breast cancer
*RIPK1* receptor interacting serine/threonine kinase 1	603453	Autoinflammation with episodic fever and lymphadenopathy (618852)	AD	Recurrent fever in early infancy, lymphadenopathy, hepatosplenomegaly
		Immunodeficiency 57 with autoinflammation (618108)	AR	Immunodeficiency, hypogammaglobulinemia
*SERPINB6* serpin family B member 6	173321	Deafness, autosomal recessive 91 (613453)	AR	Progressive, age-related sensorineural hearing loss
*TFAP2A* transcription factor AP-2 alpha	107580	Branchiooculofacial syndrome (113620)	AD	Branchial cleft sinus defects, ocular anomalies such as microphthalmia and lacrimal duct obstruction, and a dysmorphic facial appearance including cleft or pseudocleft lip/palate
*TUBB2A* tubulin beta 2A class IIa	615101	Cortical dysplasia, complex, with other brain malformations 5 (615763)	AD	Mild to severe mental retardation, strabismus, axial tension and spasm
*TUBB2B* tubulin beta 2B class IIb	612850	Cortical dysplasia, complex, with other brain malformations 7 (610031)	AD	Growth retardation, epilepsy, cerebellar hypoplasia, dysplasia of corpus callosum

In summary, CNV-seq is valuable for prenatal diagnosis and provides helpful genetic guidance for families who are at a high-risk of 5p deletion syndrome. In the present study, we redefined relevant region in 5p deletion syndrome, and proposed that the [GRCh37] 5p15.31:7,700,000_8,740,000 region was responsible for the cat-like cry. The importance of parental validation for variant pathogenicity assessment was also demonstrated. Furthermore, we successfully identified the breakpoints in family 3 by performing WGS, confirming the potential of WGS in detecting chromosomal translocations (10, 37). However, more cases are required to confirm the application of WGS in prenatal diagnosis, and further studies should be implemented on gene function to elucidate the genotype-phenotype correlation. In addition, parallel testing with multiple approaches, albeit at a higher cost, would be an appropriate option for prenatal diagnosis in order to shorten the detection cycle and allow for timely follow-up interventions. Our study may pave the way for further research on the prevention, diagnosis, and treatment of related diseases. Interactive confirmation of more case series would be beneficial for genetic counseling, choice of diagnostic methods, as well as pathogenic analysis of variants.

## Data Availability Statement

The datasets presented in this study can be found in online repositories. The names of the repository/repositories and accession number(s) can be found below: The datasets of CNV-seq generated and analysed during the current study are available in the Sequence Read Archive repository (https://dataview.ncbi.nlm.nih.gov/object/PRJNA779115?review er=vjtb6iiga3mi0209bocdcqdadt).

## Ethics Statement

The studies involving human participants were reviewed and approved by the Chinese PLA General Hospital Medical Ethics Committee (S2016-120-02). Written informed consent to participate in this study was provided by the participants’ legal guardian/next of kin.

## Author Contributions

RH designed the verification experiments and conducted data analysis. GC and PL wrote the manuscript. JW and GZ performed the sample collection. YZ revised the article. All authors contributed to the article and approved the submitted version.

## Conflict of Interest

The authors declare that the research was conducted in the absence of any commercial or financial relationships that could be construed as a potential conflict of interest.

## Publisher’s Note

All claims expressed in this article are solely those of the authors and do not necessarily represent those of their affiliated organizations, or those of the publisher, the editors and the reviewers. Any product that may be evaluated in this article, or claim that may be made by its manufacturer, is not guaranteed or endorsed by the publisher.

## Footnotes

1
http://bio-bwa.sourceforge.net/

2
http://www.broadinstitute.org/gatk/

## Supplementary Material

Supplementary Figure 1 The diagnosis protocol of work up for 5p deletion syndrome.

Supplementary Figure 2 STRs results of the amniotic fluid (III-2) in family 2 using QF-PCR. Peaks of six markers (AMEL, XHPRT, X22, DXS6803, TAF9L, and DXYS267) were shown in dashed boxes, corresponding fragment size and peak height were presented in solid boxes.
