# Supplementary figures and images for: Copy Number Variation Analysis of 5p Deletion Provides Accurate Prenatal Diagnosis and Reveals Candidate Pathogenic Genes
Source: Front Med (Lausanne). 2022 Jul 14;9:883565. doi: 10.3389/fmed.2022.883565 (PMC9329539; doi:10.3389/fmed.2022.883565)

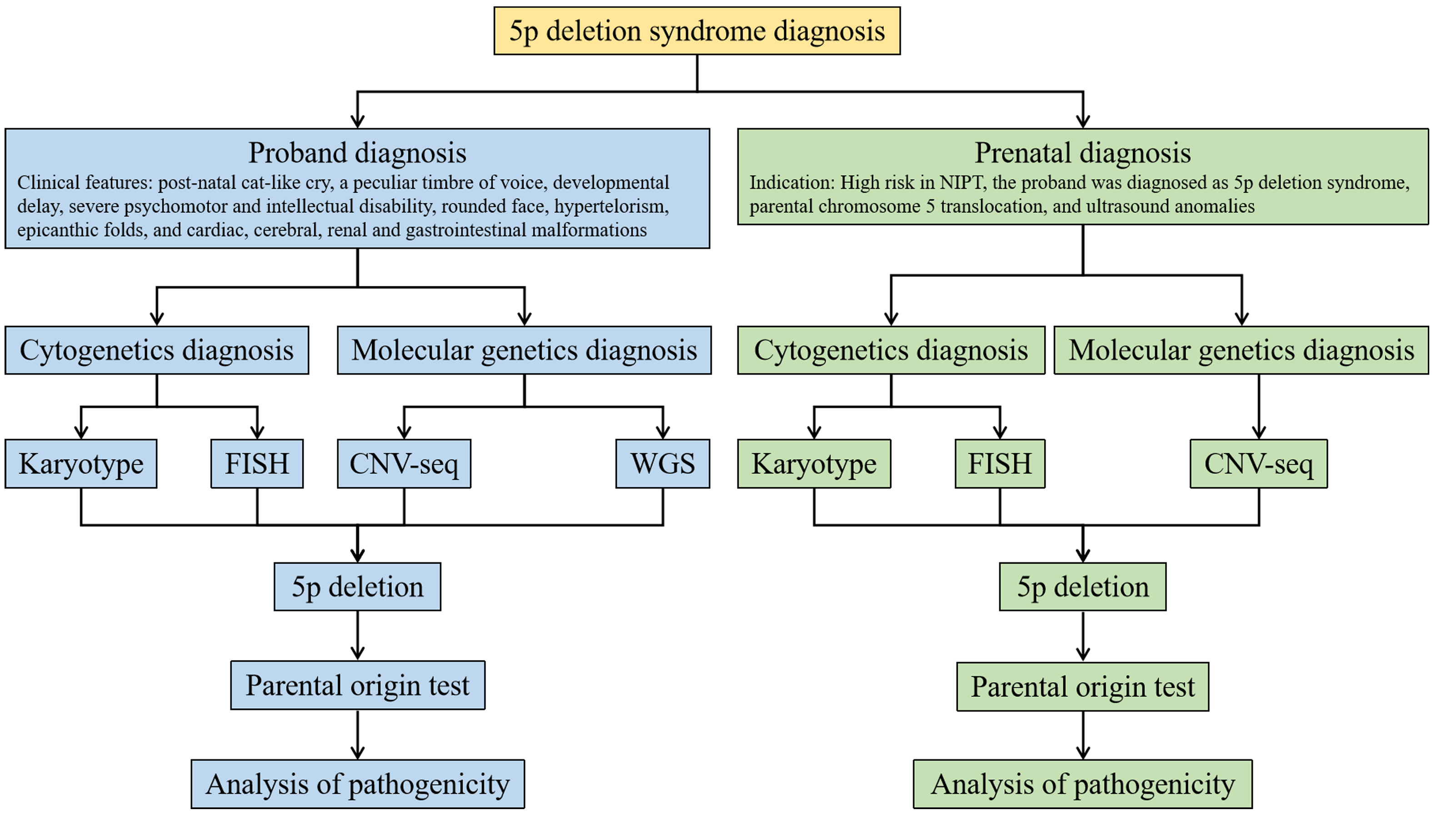

Supplement: Supplementary Figure 1 — The diagnosis protocol of work up for 5p deletion syndrome. [file Image_1.JPEG]

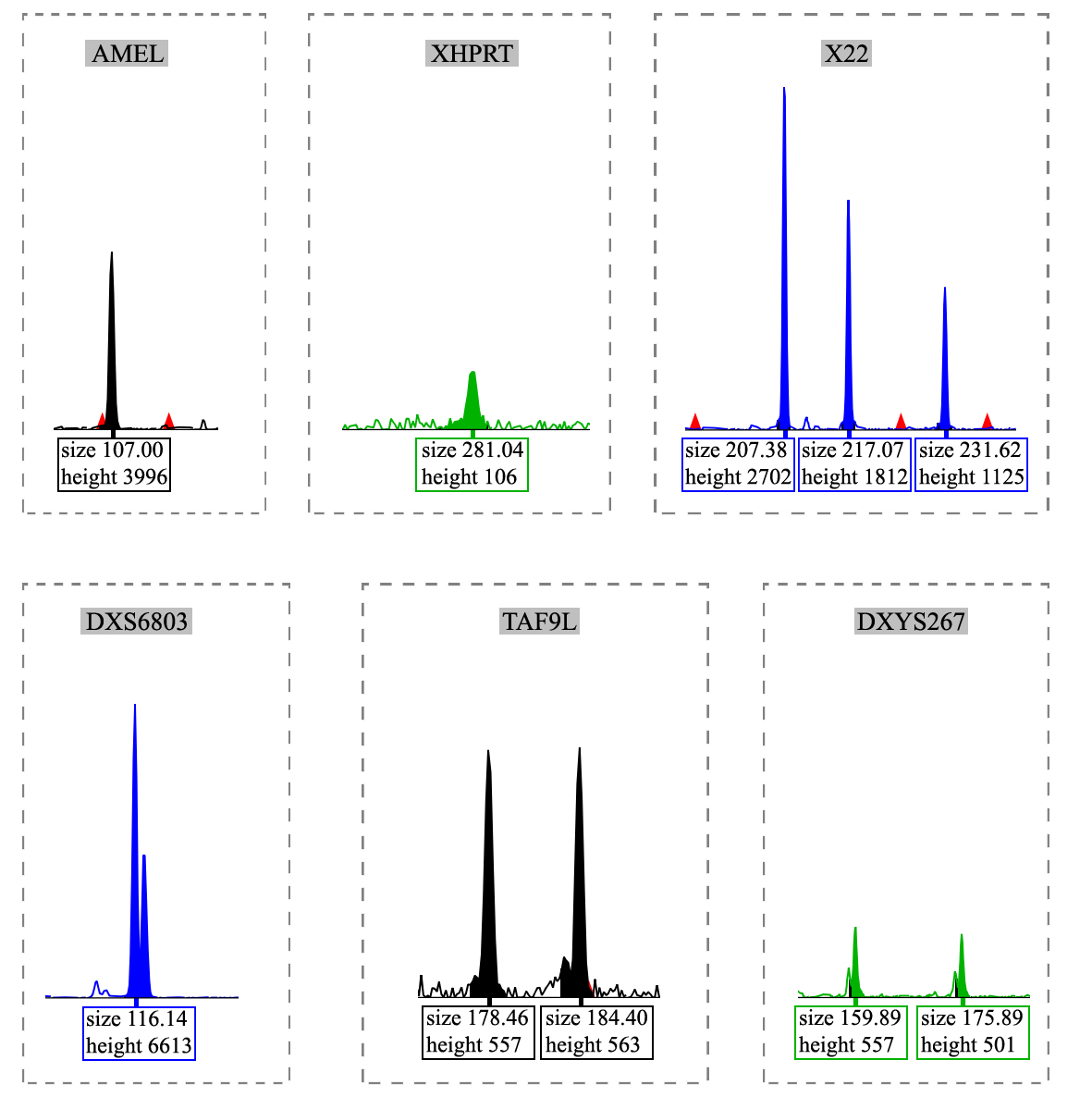

Supplement: Supplementary Figure 2 — STRs results of the amniotic fluid (III-2) in family 2 using QF-PCR. Peaks of six markers (AMEL, XHPRT, X22, DXS6803, TAF9L, and DXYS267) were shown in dashed boxes, corresponding fragment size and peak height were presented in solid boxes. [file Image_2.JPEG]
